# Chungsimyeonja-eum inhibits inflammatory responses in RAW 264.7 macrophages and HaCaT keratinocytes

**DOI:** 10.1186/s12906-015-0902-2

**Published:** 2015-10-16

**Authors:** Hye-Sun Lim, Kim Yeji, Chang-Seob Seo, Sae-Rom Yoo, Seong-Eun Jin, Hyeun-Kyoo Shin, Soo-Jin Jeong

**Affiliations:** K-herb Research Center, Korea Institute of Oriental Medicine, Daejeon, 34054 Republic of Korea; Division of Allergy and Chronic Respiratory Diseases, Center for Biomedical Sciences, Korea National Institute of Health, Chungcheongbuk-do, 28159 Republic of Korea; Korean Medicine Convergence Research Division, Korea Institute of Oriental Medicine, Daejeon, 34054 Republic of Korea; Korean Medicine Life Science, University of Science & Technology, 217 Gajeong-ro Yuseong-gu, Daejeon, 34113 Republic of Korea

**Keywords:** Chungsimyeonja-eum, Inflammation, Cytokine, Chemokine, Macrophage, Keratinocyte

## Abstract

**Background:**

Chungsimyeonja-eum (CSYJE) is an herbal prescription used in traditional Oriental medicine for treating cerebral infarction by reducing ischemic damage. However, the effects of CSYJE on inflammation have not been verified scientifically.

**Methods:**

Anti-inflammatory effects of CSYJE was investigated to dertermine the inhibitory effects of CSYJE against inflammation using RAW 264.7 mouse macrophages and HaCaT human keratinocytes. To measure the effects of CSYJE on inflammatory mediators and cytokines/chemokines, we used the following methods: cell viability assay, enzyme-linked immunosorbent assay (ELISA), western blotting, immunocytochemistry. RAW 264.7 cells were pretreated with CSYJE (250, 500, or 1000 μg/mL) for 4 h and treated with lipopolysaccharide (LPS) for additional 20 h. HaCaT cells were stimulated with tumor necrosis factor alpha (TNF-α) and interferon gamma (IFN-γ) (TI), and CSYJE (125, 250, or 500 μg/mL) for 24 h.

**Results:**

CSYJE suppressed the production of nitric oxide (NO, IC_50_ 1000 μg/mL), prostaglandin E_2_ (PGE_2_, IC_50_ = 12.1 μg/mL), and interleukin (IL)-6 (IC_50_ = 248 μg/mL) in LPS-stimulated RAW 264.7 cells. CSYJE suppressed the effects of TI on the production of thymus and activation-regulated chemokine (TARC, IC_50_ = 330.2 μg/mL), macrophage-derived chemokine (MDC/CCL22, IC_50_ = 52.5 μg/mL), regulated on activation, normal T-cell expressed and secreted (RANTES/CCL5, IC_50_ = 372.9 μg/mL), and IL-8 (IC_50_ = 345.1 μg/mL) in HaCaT cells. CSYJE inhibited TI-stimulated STAT1 phosphorylation in a dose-dependent manner and nuclear translocation at 500 μg/mL in HaCaT cells.

**Conclusion:**

Our results suggest a possible therapeutic application of CSYJE for treating inflammatory diseases.

## Backgrounds

Inflammation constitutes multiple processes, characterized by the infiltration of inflammatory cells, such as Th2-type cells, eosinophils, mast cells, and macrophages. Of these activated cells, macrophages play a central role in managing many different pathological immune phenomena such as the over-production of inflammatory mediators. Various mediators, such as nitric oxidase, prostaglandins, and inflammatory cytokines such as interleukin (IL)-6, IL-1β and tumor necrosis factor (TNF)-α, and others induced by macrophages have important roles in inflammatory diseases [1–3].

Migration of inflammatory cells is regulated by chemokines. These are small proteins released from various cell types, which regulate the traffic of immune cells to site of inflammation or infection. Various inflammatory cytokines stimulate the expression of inflammatory chemokines [4, 5]. Among them, thymus and activation-regulated chemokine (TARC/CCL17), macrophage-derived chemokine (MDC/CCL22), and regulated on activation, normal T-cell expressed and secreted (RANTES/CCL5) are typical inflammatory chemokines and ligands for CC chemokine receptor 4 (CCR4), which is predominantly expressed on keratinocytes, Th2 lymphocytes, and basophils [6, 7]. In addition, IL-8 is another inflammatory mediator involved in allergic responses that is closely associated with the severity of chronic inflammation [8].

Extracellular binding of cytokines induces activation of the intracellular Janus kinase (JAK) that phosphorylates a specific tyrosine residue in signal transducer and activator of transcription (STAT) proteins [9], which are involved in the development and function of the immune system [10]. There are seven mammalian STAT family members that have been identified: STAT1, STAT2, STAT3, STAT4, STAT5 (STAT5A and STAT5B) and STAT6. In particular, STAT1 is a crucial molecule for the interferon (IFN)/cytokine-signaling pathways [11]. Thus, IFN-γ stimulates JAK phosphorylation, which in turn induces STAT1 phosphorylation [12]. These events eventually increase the production of chemokines including TARC, MDC, and RANTES [13, 14].

The traditional herbal formula *Chungsimyeonja*-*eum* (CSYJE) comprises nine medicinal herbs and has traditionally been used in the treatment of heart and brain diseases in Oriental medicine. Previous research reported that the water extract of CSYJE has cytoprotective effect against glutamate-induced C6 glial cell death [15]. Other studies showed that CSYJE protects against ischemic stroke and has anti-stress activity [16, 17]. However, there has been no investigation focusing on the inflammatory effects of CSYJE. Therefore, we investigated the effects and action mechanisms of CSYJE on the inflammatory mediators in RAW 264.7 macrophages and HaCaT keratinocytes.

## Methods

### Preparation of CSYJE water extract

The 9 crude herbal medicines forming CSYJE were purchased from Kwangmyungdang Medicinal Herbs (Ulsan, Korea). The origin of 9 herbal medicines was confirmed taxonomically by Professor Je-Hyun Lee, Dongguk University, Gyeongju, Republic of Korea. A voucher specimen (2012–KE43–1 ~ KE43–9) has been deposited at K-herb Research Center, Korea Institute of Oriental Medicine. For preparation of CSYJE water decoction, each herbal medicine was mixed as Table 1 (total weight = 5.0 kg, about 156.9 times of composition of single dose) and extracted in distilled water at 100 °C for 2 h under pressure (98 kPa) using an electric extractor (COSMOS-660; Kyungseo Machine Co., Incheon, Korea). The extract solution was filtrated using a standard sieve (No. 270, 53 μm; Chung Gye Sang Gong Sa, Seoul, Korea) and freeze-dried to give a powder sample. The yield of CSYJE extract was 13.0 % (651.4 g).

**Table 1:** Herbal composition of a single dose of CSYJE

Latin name	Scientific name	Amount (g)	Origin
Nelumbinis Semen	*Nelumbo nucifera* Gaerhner	7.500	China
Poria Sclerotium	*Poria cocos* Wolf	3.750	Pyeongchang, Korea
Ginseng Radix	*Panax ginseng* C. A. Meyer	3.750	Yeongju, Korea
Astragali Radix	*Astragalus membranaceus* Bunge	3.750	Jecheon, Korea
Scutellariae Radix	*Scutellaria baicalensis* Georgi	2.625	Gurye, Korea
Plantaginis Semen	*Plantago asiatica* L.	2.625	China
Liriope Tuber	*Liriope platyphylla* Wang et Tang	2.625	Miryang, Korea
Lycii Radicis Cortex	*Lycium chinense* Miller	2.625	China
Glycyrrhizae Radix et Rhizoma	*Glycyrrhiza uralensis* Fischer	2.625	China
Total		1.875	

### High-performance liquid chromatography (HPLC) analysis of CSYJE

Quantitative analysis of the CSYJE sample was performed using a Shimadzu LC-20A Prominence HPLC system (Shimadzu Co., Kyoto, Japan) consisting of a solvent delivery unit, an on-line degasser, a column oven, an autosampler, and a photo diode array (PDA) detector. The data were acquired and processed by LabSolution software (Version 1.24 SP1). A SunFire C_18_ column (250 × 4.6 mm; particle size 5 μm, Waters, Milford, MA, USA), which was maintained at 40 °C, was used as the stationary phase and the mobile phases were consisted of 0.1 % (v/v) formic acid in water (A) and 0.1 % (v/v) formic acid in acetonitrile (B). The elution conditions were as follows: 10–60 % B for 0–30 min, 60–100 % B for 30–40 min, 100 % B for 40–45 min, and 100–10 % B for 45–50 min. The flow-rate was 1.0 mL/min and injection volume was 10 μL. For HPLC analysis of CSYJE, lyophilized 200 mg of CSYJE extract was dissolved in 20 mL of distilled water and then the solution was diluted to 10-fold for quantitative analysis of baicalin and wogonoside. Each solution was filtered through a SmartPor GHP 0.2 μm syringe filter (Woongki Science, Seoul, Korea) before HPLC injection.

### Cell cultures

Murine macrophage RAW 264.7 and human keratinocyte HaCaT were obtained from the American Type Culture Collection (Rockville, MD) and CLS Cell Lines Service GmbH (Eppelheim, Baden-Württemberg, Germany), respectively. The cells were cultured in Dulbecco’s modified Eagle’s medium (Gibco Inc., Grand Island, NY) supplemented with 5.5 % (for RAW 264.7) or 10 % (for HaCaT) heat-inactivated fetal bovine serum (Gibco Inc.), penicillin (100 U/mL), and streptomycin (100 μg/mL) in a 5 % CO_2_ incubator at 37 °C.

### Cytotoxicity assay

Cell viability was assessed using a Cell Counting Kit-8 assay (CCK-8 from Dojindo, Kumamoto, Japan) according to the manufacturer’s instructions. RAW 264.7 cells (3 × 10^3^ cells/well) and HaCaT cells (1 × 10^3^ cells/well) were incubated in 96-well plates with various concentrations of the CSYJE for 24 h. The CCK-8 reagent was added to each well, followed by incubation for an additional 4 h, and the absorbance was measured at 450 nm using a Benchmark plus microplate reader (Bio-Rad Laboratories, Hercules, CA). The percentage of viable cells was calculated using the following equation: cell viability (%) = (mean absorbance in test well/mean absorbance in control well) × 100.

### Measurement of nitric oxide (NO), prostaglandin E_2_ (PGE_2_), and IL-6 production

RAW 264.7 cells were plated at a density of 2.5 × 10^5^ cells/well in 48-well plates and incubated overnight. Cells were pretreated with various concentrations of CSYJE for 4 h and then treated with LPS (1 μg/mL) for an additional 20 h. The supernatants were collected and analyzed for the levels of NO (Griess Reagent System; Promega Corp., Madison, WI), and PGE_2_ and IL-6 using enzyme-linked immunosorbent assay (ELISA) kits (Cayman Chemical Co., Ann Arbor, MI for PGE_2_, and R&D Systems, Minneapolis, MN) according to the manufacturers’ protocols.

### Measurement of chemokine production

HaCaT cells (1 × 10^6^ cells/well) were cultured in 6-well plates. After reaching confluency, the cells were washed and treated with CSYJE in 1 mL of serum-free medium containing tumor necrosis factor-α (TNF-α) and interferon-γ (IFN-γ) (TI, each 10 ng/mL; R&D Systems Inc., Minneapolis, MN, USA) for 24 h. The production of TARC, MDC, RANTES, and IL-8 was determined using commercial ELISA kits (R&D Systems Inc.).

### Western blotting

HaCaT cells were treated with a nontoxic concentration of CSYJE for 1 h, and then incubated in the presence of TI for 30 min. The cells were collected by centrifugation, washed twice with PBS, and suspended in an extraction lysis buffer (Sigma-Aldrich, St. Louis, MO) containing protease inhibitors. The protein concentration was determined using a protein assay reagent (Bio-Rad Laboratories, Hercules, CA) according to the manufacturer’s instructions. Equal amounts of total protein (30 μg) were resolved by 10 % sodium dodecyl sulfate-polyacrylamide gel electrophoresis (SDS-PAGE) and transferred to a nitrocellulose membrane. The membrane was incubated with blocking solution [5 % skim milk in Tris-buffered saline containing Tween 20 (TBST)], followed by an overnight incubation at 4 °C with the appropriate primary antibody. The following primary antibodies and dilution were used: anti-β-actin (1:1000 dilution; Cell Signaling, Danvers, MA), anti-STAT1, and anti-phospho-STAT1 (1:1000 dilution; Abcam, Cambridge, UK). The membranes were washed three times with TBST, and then incubated with a 1:3000 dilution of a horseradish peroxidase (HRP)-conjugated secondary antibody (Jackson ImmunoResearch, West Grove, PA) for 1 h at room temperature. The membranes were again washed three times with TBST, and then developed using an enhanced chemiluminescence (ECL) kit (Thermo scientific, Rockford, IL). Image capture was performed using Chemi-Doc (Bio-Rad).

### Immunofluorescence staining

HaCaT cells were seeded onto glass coverslips and incubated with TI in the absence or presence of CSYJE (500 μg/mL) for 30 min. The cells were fixed in 4 % paraformaldehyde and 100 % acetone, blocked in 0.5 % bovine serum albumin, and incubated with anti-STAT1 antibody (Cell Signaling, Danvers, MA) for 1 h at room temperature. Then, fluorescein isothiocyanate (FITC)-conjugated anti-rabbit immunoglobulin G (IgG) antibody (Invitrogen, Carlsbad, CA) was used as a secondary antibody. The immunostained cells were mounted with medium containing 4′6-diamidino-2-phenylindole (DAPI) and visualized using an Olympus FLUOVIEW FV10i confocal microscope (Tokyo, Japan).

### Statistical analysis

The data are expressed as the mean ± SEM. Data were analyzed using one-way analysis of variance and Dunnett’s multiple comparisons test. Results with a *P* value < 0.05 were considered to be statistically significant.

## Results

### Identification and quantification of the marker components in CSYJE

**Fig. 1:**
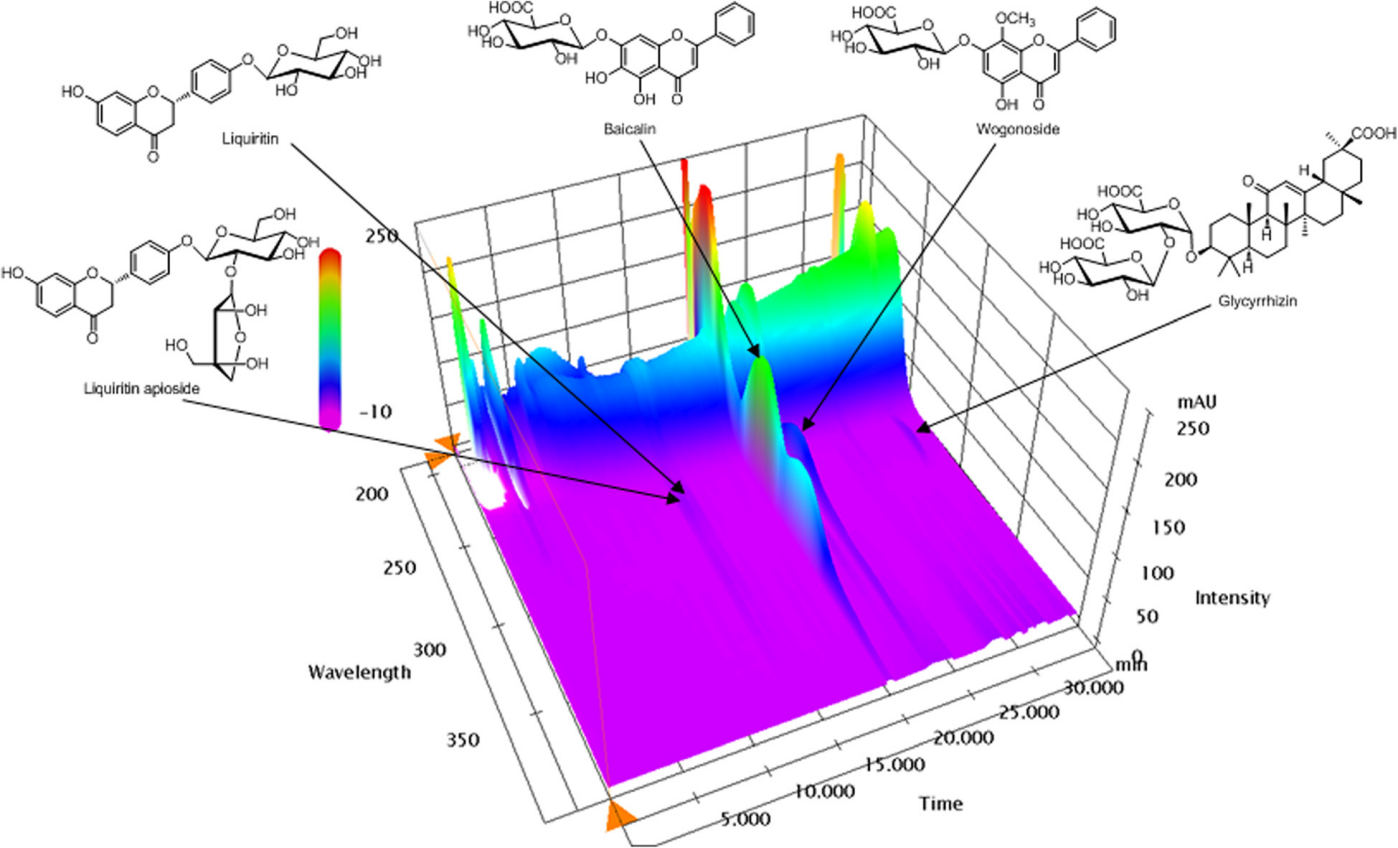
Three-dimensional chromatogram of CSYJE by HPLC-PDA. The retention times of the five marker compounds, liquiritin apioside, liquiritin, baicalin, wogonoside, and glycyrrhizin, were appeared at approximately 13.86, 14.26, 20.00, 23.18, and 28.51 min, respectively

Calibration curves of the five marker components showed good linearity with correlation coefficient (*r*^2^) ≥ 0.9998 in the different concentration ranges. Using optimized chromatography conditions, Three-dimensional chromatogram was obtained using HPLC–PDA detector and the five marker compounds were eluted within 35 min (Fig. 1). The amounts of the five marker compounds, liquiritin apioside, liquiritin, baicalin, wogonoside, and glycyrrhizin, were 1.43 ± 0.02, 2.00 ± 0.03, 22.52 ± 0.36, 7.11 ± 0.02, and 2.49 ± 0.02 mg/g, respectively.

### Effects of CSYJE on cell viability of RAW 264.7 mouse macrophages and HaCaT human keratinocytes

**Fig. 2:**
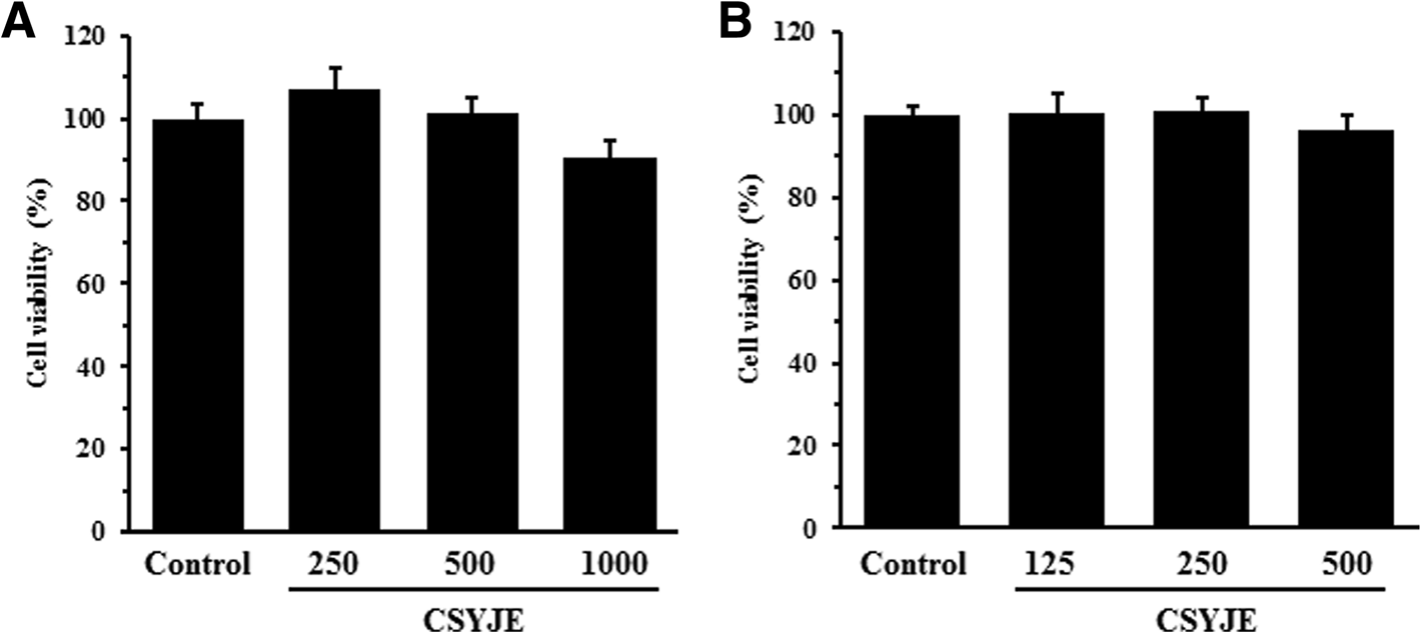
Cytotoxicity of CSYJE against RAW 264.7 cells or HaCaT cells. RAW 264.7 (**a**) or HaCaT (**b**) cells were seeded onto 96-well plates and treated with various concentrations of CSYJE for 24 h. Cell viability (%) was assessed using a CCK-8 assay. The values are expressed as the mean ± SEM of three independent experiments

To examine the effect of CSYJE on cell viability, RAW 264.7 cells and HaCaT cells were treated with various concentration ranging from 250 to 1000 μg/mL and 125 to 500 μg/mL of CSYJE for 24 h, respectively. As shown in Fig. 2, the survival of RAW 264.7 cells and HaCaT cells were not affected by exposure to concentration of CSYJE compared to that of the controls. Therefore, nontoxic concentrations (250, 500, or 1000 μg/mL in RAW 264.7 cells; 125, 250, or 500 μg/mL in HaCaT cells) of CSYJE were used for the subsequent experiments.

### CSYJE inhibits production of pro-inflammatory mediators in lipopolysaccharide (LPS)-stimulated RAW 264.7 cells

**Fig. 3:**
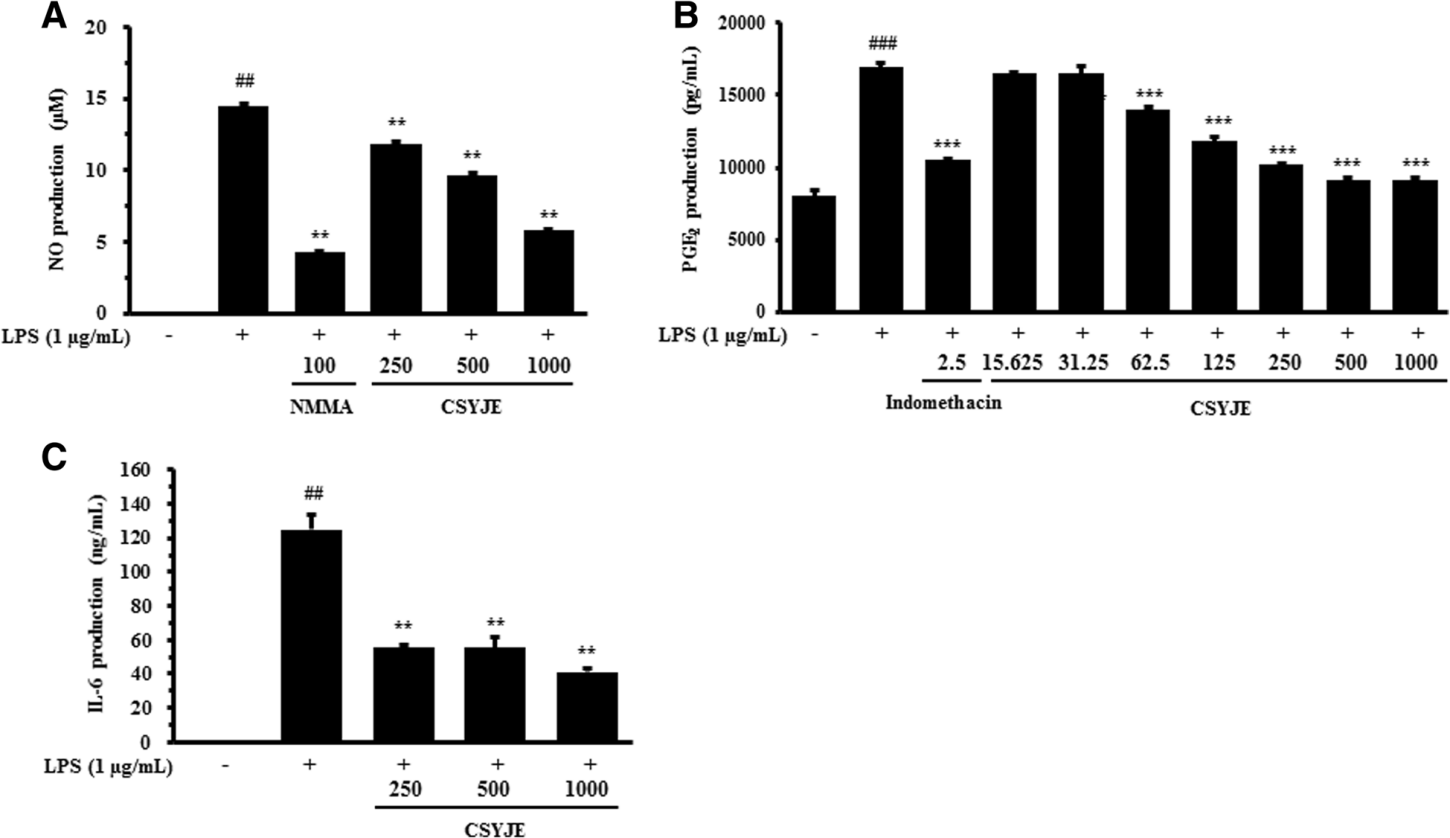
Effects of CSYJE on LPS-stimulated production of pro-inflammatory mediators in RAW 264.7 cells. Levels of NO (**a**), PGE_2_ (**b**), and IL-6 (**c**) were measured in the culture supernatants from cells treated with CSYJE and LPS. Cells were pretreated with CSYJE (250, 500, or 1000 μg/mL) for 4 h and LPS (1 μg/mL) for additional 20 h for NO, PGE_2_, and IL-6 assays. Cells were pretreated with CSYJE (250, 500, or 1000 μg/mL for NO and IL-6 assays, and 15.625, 31.25, 62.5, 125, 250, 500, or 1000 μg/mL for PGE_2_ assay) for 4 h and LPS (1 μg/mL) for additional 20 h for PGE_2_ assay. L-N-methylarginine (L-NMMA; (100 μM) and indomethacin (2.5 ng/mL) were used as positive control drugs. The bar graphs represent the means from three independent experiments. ^##^ *P* < 0.01 vs vehicle control cells; and **P < 0.01 vs LPS-treated cells

To determine the effects of CSYJE on NO and PGE_2_ production following LPS stimulation, RAW 264.7 cells were pretreated with various concentrations of CSYJE (0, 250, 500, or 1000 μg/mL) for 4 h and then stimulated with LPS (1 μg/mL) for an additional 20 h. CSYJE suppressed LPS-stimulated NO production in a dose-dependent manner (Fig. 3a). LPS significantly stimulated NO production by RAW 264.7 cells (14.42 ± 0.24 μM). By contrast, CSYJE significantly decreased NO production to 9.67 ± 0.09 μM (*P* < 0.01) at 500 μg/mL and to 5.76 ± 0.15 μM (*P* < 0.01) at a dose of 1000 μg/mL. PGE_2_ production was higher in LPS-stimulated RAW 264.7 cells than in untreated control cells, whereas CSYJE dose-dependently inhibited PGE_2_ production by LPS stimulation (Fig. 3b). Furthermore, LPS treatment dramatically increased IL-6 level up to 123.96 ng/mL compared with untreated control. In contrast, CSYJE significantly inhibited IL-6 production by 54.82, 55.31, and 66.89 % at 250, 500, and 1000 μg/mL, respectively (Fig. 3c).

### CSYJE inhibits chemokine production in TNF-α and IFN-γ-stimulated HaCaT cells

**Fig. 4:**
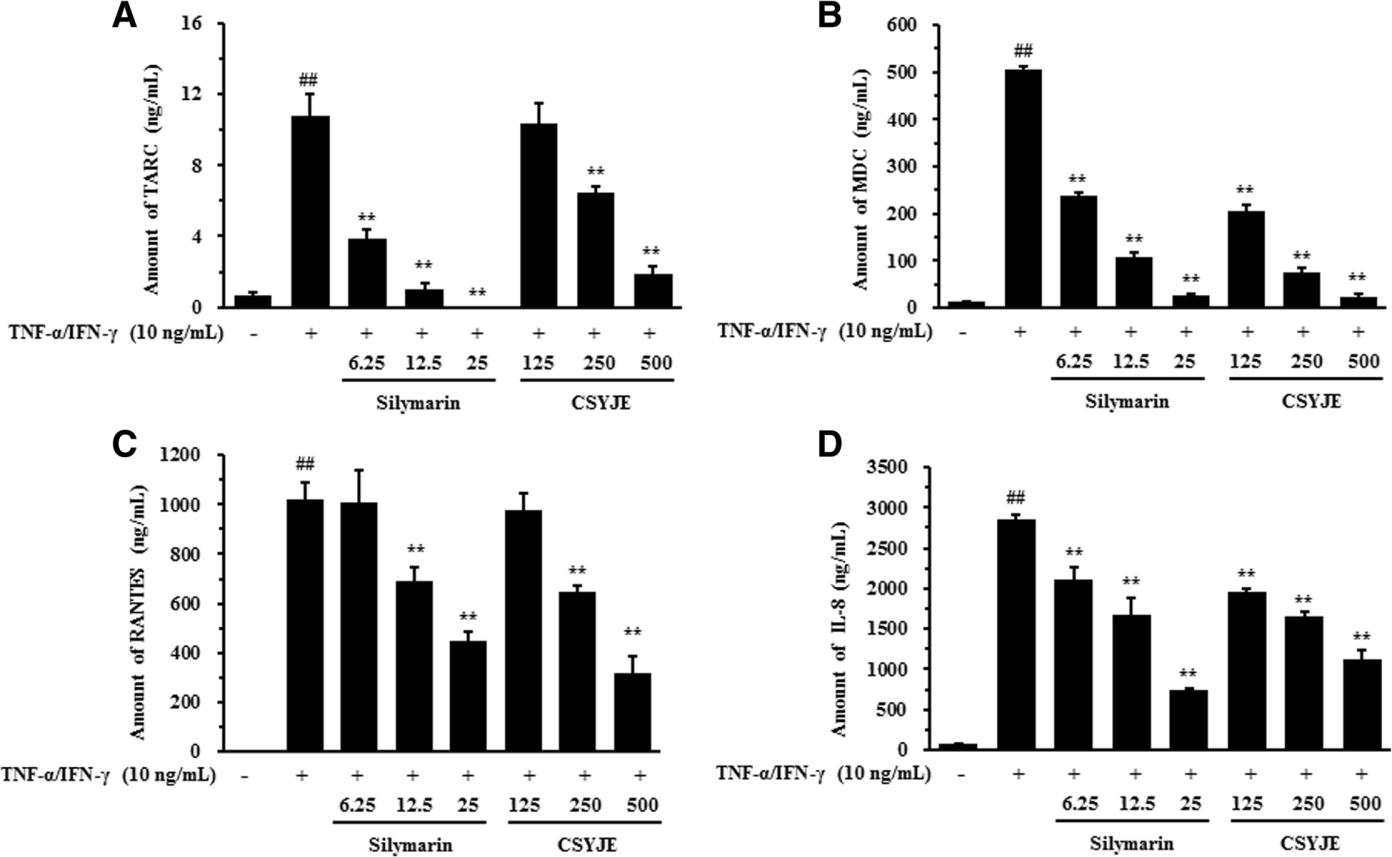
Effects of CSYJE on TNF-α and IFN-γ-stimulated productions of chemokines in HaCaT cells. Levels of TARC (**a**), MDC (**b**), RANTES (**c**), and IL-8 (**d**) were measured using the culture supernatants from cells treated with CSYJE (125, 250, or 500 μg/mL), and TNF-α and IFN-γ (each 10 ng/mL, TI) for 24 h. Silymarin (6.25, 12.5, or 25 μg/mL) was used as a positive control. Values were expressed as mean ± SEM of three independent experiments. ^##^ *P* < 0.01 vs vehicle control cells; and **P < 0.01 vs TI-treated cells

The effects of CSYJE on TARC, MDC, RANTES, and IL-8 production levels were assessed in TI-stimulated HaCaT cells. As shown in Fig. 4a, HaCaT cells treated with TI increased the TARC level (10.78 ± 1.21 ng/mL, *P* < 0.01) compared with the vehicle-treated cells (0.65 ± 0.18 ng/mL). By contrast, CSYJE suppressed TI-stimulated TARC production in a dose-dependent manner. The levels were reduced by 6.44 ± 0.35 ng/mL (*P* < 0.01) and 1.88 ± 0.45 ng/mL (*P* < 0.01) using 250 and 500 μg/mL, respectively. MDC production in TI-treated cells was also increased (506.70 ± 7.27 ng/mL, *P* < 0.01) compared with vehicle-treated cells, but the levels were reduced significantly after treatment with CSYJE treatment (205.25 ± 16.80 ng/mL at 125 μg/mL, *P* < 0.01; 74.15 ± 11.59 ng/mL at 250 μg/mL, *P* < 0.01; and 24.34 ± 4.69 ng/mL at 500 μg/mL, *P* < 0.01) (Fig. 4b). TI-treated cells produced significantly more RANTES (1019.80 ± 66.97 ng/mL, *P* < 0.01) compared with the vehicle-treated cells. The TI-induced increase in RANTES production was significantly inhibited by CSYJE (644.18 ± 29.79 ng/mL at 250 μg/mL, *P* < 0.01; and 319.94 ± 68.15 ng/mL at 500 μg/mL, *P* < 0.01) (Fig. 4c). In addition, the level of IL-8 (2848.74 ± 98.83 ng/mL, *P* < 0.01) was also significantly reduced by CSYJE in a dose-dependent manner (1648.33 ± 56.81 ng/mL at 250 μg/mL, *P* < 0.01; and 1126.23 ± 38.02 ng/mL at 500 μg/mL, *P* < 0.01) (Fig. 4d). The inhibitory effects of CSYJE were similar to those of silymarin, which was used as a positive control.

### CSYJE inhibits STAT1 phosphorylation in TNF-α and IFN-γ-stimulated HaCaT cells

**Fig. 5:**
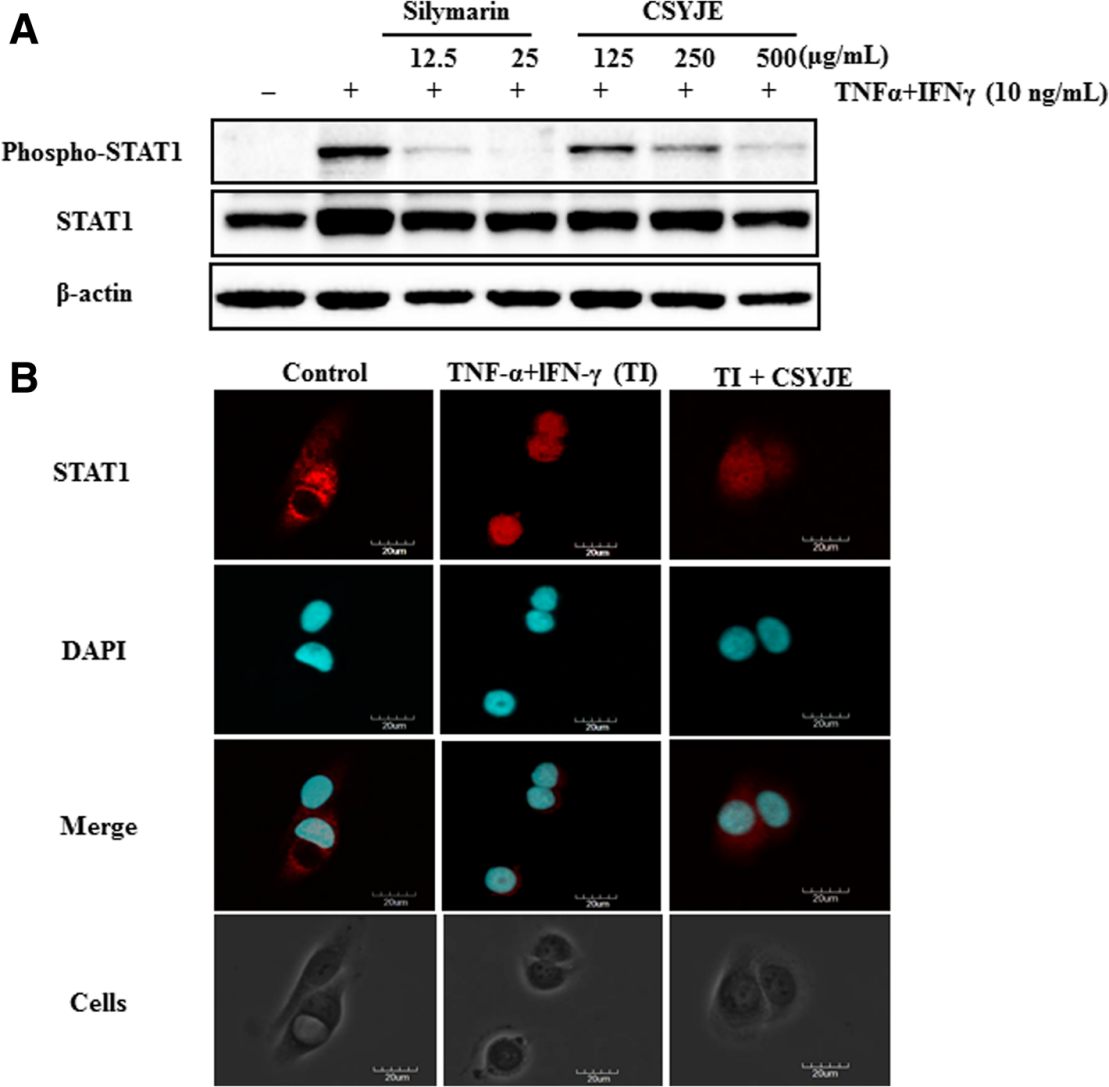
Changes in TNF-α and IFN-γ-stimulated STAT1 phosphorylation in CSYJE-treated HaCaT cells. **a** The expression of STAT1 phosphorylation was measured using cells cotreated with CSYJE, and TNF-α and IFN-γ (each 10 ng/mL, TI) for 24 h. Silymarin (12.5, or 25 μg/mL) was used as a positive control. **b** Cellular localization of STAT1 was examined by immunofluorescence staining. Cells were co-treated with CSYJE (500 μg/mL), and TI for 30 min. The cells were fixed in 4 % (v/v) methanol-free formaldehyde solution (pH 7.4), stained with anti-STAT1 (red). The stained cells were mounted in medium containing DAPI (blue) and visualized under an Olympus FLUOVIEW FV 10i confocal microscope

TI-stimulated HaCaT cells showed markedly increased STAT1 phosphorylation compared with non-stimulated HaCaT cells. Treatment with silymarin reduced the level of STAT1 phosphorylation compared with TI stimulation alone. Treatment with CSYJE showed a dramatic reduction in STAT1 phosphorylation in a dose-dependent manner (Fig. 5a). Consistent with the results of western blotting, immunofluorescence staining revealed that CSYJE inhibited the TI-induced nuclear localization of STAT1 in HaCaT cells (Fig. 5b).

## Discussion

CSYJE, a traditional herbal fomula, is composed of 9 medicinal herbs such as Nelumbinis Semen, Poria Sclerotium, Ginseng Radix, Astragali Radix, Scutellariae Radix, Plantaginis Semen, Liriope Tuber, Lycii Radicis Cortex, and Glycyrrhizae Radix et Rhizoma and has been used to treat neuropsychiatric disease, stroke, renal tuberculosis, stomatitis, pyelitis, and cystitis in Korea [18]. The major components of the 9 herbal medicines are known as follows: flavonoids (e.g. isoquercitrin and quercetin-3-*O*-glucuronide) from Nelumbinis Semen [14], triterpenoids (e.g. pachymic acid and dehydropachymic acid) from Poria Sclerotium [19, 20], triterpenoids (e.g. ginsenoside Rb1 and Rb1) from Ginseng Radix [21], flavonoids (e.g. formononetin and calycosin-7-*O*-glucoside) from Astragali Radix [22], flavonoids (e.g. baicalin and wogonoside) from Scutellariae Radix [23], iridoids (e.g. genoiposide and genipsidic acid) form Plantaginis Semen [24, 25], steroid saponins (e.g. spicatoside A and B) form Liriope Tuber [26], phenylpropanoid (chlorogenic acid) and flavonoid (e.g. rutin) form Liriope Tuber [27], and triterpene saponin (e.g. glycyrrhizin) and flavonoids (e.g. liquiritin and liquiritigenin), from Glycyrrhizae Radix et Rhizoma [28]. Among those constituents, we tried to analyze 19 compounds, such as hyperoside, isoquercitrin, pachymic acid, ginsenoside Rb1, ginsenoside Rg1, formononetin, baicalin, baicalenin, wogonoside, wogonin, geniposide, geniposidic acid, spicatoside A, chlorogenic acid, rutin, liquiritigenin, liquiritin, liquiritin apioside, and glycyrrhizin using optimized HPLC–PDA method in CSYJE sample. Consequently, the main components of Scutellariae Radix Glycyrrhizae Radix et Rhizoma, liquiritin apioside, liquiritin, baicalin, wogonoside, and glycyrrhizin were detected. However, other components were not detected in this sample. Finally, baicalin (22.52 ± 0.36 mg/g), marker compound of Scutellariae Radix, was detected as the major compound in CSYJE extract.

In the present study, we found that CSYJE inhibited the actions of various inflammatory mediators. CSYJE inhibited production of the pro-inflammatory mediators NO and PGE_2_, and cytokine IL-6 in LPS-stimulated macrophages. CSYJE also inhibited the production of the chemokines TARC, MDC, RANTES, and IL-8, and suppressed STAT1 activation in TI-treated keratinocytes.

Macrophages, as innate immune cells, initiate inflammation and the immune response [29]. When challenged with LPS, macrophages are activated and release various pro-inflammatory factors, excessive release of which can result in extensive tissue damage and pathological changes. NO, an important cellular signaling molecule and pro-inflammatory mediator produced by inducible nitric oxide synthase (iNOS), plays a key role in the pathogenesis of inflammation caused by its overproduction in abnormal situations [30]. PGE_2_ is another important pro-inflammatory factor regulated by cyclooxygenase-2 (COX-2). Its expression is elevated in LPS-stimulated macrophages, and is associated with many chronic inflammatory diseases including cardiovascular diseases, arthritis, inflammatory bowel disease, angiogenesis, and chronic gastric ulcers [31–33]. Macrophages also release pro-inflammatory cytokines including IL-1β, IL-6, and TNF-α, which are used to evaluate potential anti-inflammatory properties of agents against LPS-induced macrophage activation [34]. In the present study, CSYJE significantly inhibited the concentrations of NO and PGE_2_ in the supernatant in LPS-stimulated RAW 264.7 cells. Thus, CSYJE might reduce inflammatory responses by inhibiting IL-6 secretion.

Keratinocytes produce various chemokines, which are involved in the development of inflammatory diseases. TARC and MDC are produced by dendritic cells, endothelial cell and keratinocytes which bind and attract CCR4+ Th2 cells into inflammatory tissues [7]. Several studies have reported that there are high levels of TARC and MDC in the serum of patients with atopic dermatitis [35]. The severity of atopic dermatitis was significantly correlated with these chemokine levels [36]. RANTES is overexpressed in the keratinocytes of such patients. In addition, RANTES has a significant role in the inflammatory process of psoriasis. Here, we found that CSYJE inhibited TARC, MDC, RANTES, and IL-8 production in TI-stimulated HaCaT keratinocytes.

STAT1 is also a pivotal regulator of IFN-γ-induced immune responses [37]. The activated STAT1 pathway can modulate the expression of numerous inflammatory mediators, including chemokines [14]. Here we demonstrated that CSYJE inhibited the TI-induced phosphorylation and nuclear translocation of STAT1 in HaCaT cells. These data suggest that CSYJE might block the induction of chemokine production including TARC, MDC, and RANTES in TI-treated keratinocytes by inhibiting STAT1 activation.

## Conclusions

Our present study demonstrated that CSYJE inhibited the levels of NO, PGE_2_, and IL-6 in LPS-stimulated RAW 264.7 cells, and reduced the production of chemokines TARC, MDC, RANTES, and IL-8 by suppressing the activation of STAT1 in TI-stimulated HaCaT cells. Further elucidation of the signaling pathways involved in the inhibition of inflammatory mediators by CSYJE is now needed to facilitate the design of therapeutic agents for inflammatory diseases.
